# Epigenetically regulated gene expression profiles decipher four molecular subtypes with prognostic and therapeutic implications in gastric cancer

**DOI:** 10.1186/s13148-023-01478-w

**Published:** 2023-04-15

**Authors:** Siyuan Weng, Minghao Li, Jinhai Deng, Hui Xu, Yuqing Ren, Zhaokai Zhou, Libo Wang, Yuyuan Zhang, Zhe Xing, Lifeng Li, Zaoqu Liu, Xinwei Han

**Affiliations:** 1 Department of Interventional Radiology, The First Affiliated Hospital of Zhengzhou University, Zhengzhou, 450052 Henan China; 2 Interventional Treatment and Clinical Research Center of Henan Province, Zhengzhou, 450052 Henan China; 3 Department of Hepatobiliary and Pancreatic Surgery, The First Affiliated Hospital of Zhengzhou University, Zhengzhou, Henan China; 4 Richard Dimbleby Laboratory of Cancer Research, School of Cancer and Pharmaceutical Sciences, King’s College London, London, SE1 1UL UK; 5 Department of Respiratory and Critical Care Medicine, The First Affiliated Hospital of Zhengzhou University, Zhengzhou, China; 6 Department of Urologic Surgery, The First Affiliated Hospital of Zhengzhou University, Zhengzhou, 450052 Henan China; 7 Department of Neurosurgery, The Fifth Affiliated Hospital of Zhengzhou University, Henan, China; 8 Medical School, Huanghe Science and Technology University, 666 Zi Jing Shan Road, Zhengzhou, 450000 Henan China

**Keywords:** Gastric cancer, Subtype, Epigenetics, Multi-omics, Immunotherapy

## Abstract

**Background:**

Gastric cancer (GC) is one of the most common malignant tumors of the digestive tract which seriously endangers the health of human beings worldwide. Transcriptomic deregulation by epigenetic mechanisms plays a crucial role in the heterogeneous progression of GC. This study aimed to investigate the impact of epigenetically regulated genes on the prognosis, immune microenvironment, and potential treatment of GC.

**Results:**

Under the premise of verifying significant co-regulation of the aberrant frequencies of microRNA (miRNA) correlated (MIRcor) genes and DNA methylation-correlated (METcor) genes. Four GC molecular subtypes were identified and validated by comprehensive clustering of MIRcor and METcor GEPs in 1521 samples from five independent multicenter GC cohorts: cluster 1 was characterized by up-regulated cell proliferation and transformation pathways, with good prognosis outcomes, driven by mutations, and was sensitive to 5-fluorouracil and paclitaxel; cluster 2 performed moderate prognosis and benefited more from apatinib and cisplatin; cluster 3 was featured by an up-regulated ligand–receptor formation-related pathways, poor prognosis, an immunosuppression phenotype with low tumor purity, resistant to chemotherapy (e.g., 5-fluorouracil, paclitaxel, and cisplatin), and targeted therapy drug (apatinib) and sensitive to dasatinib; cluster 4 was characterized as an immune-activating phenotype, with advanced tumor stages, benefit more from immunotherapy and displayed worst prognosis.

**Conclusions:**

According to the epigenetically regulated GEPs, we developed four robust GC molecular subtypes, which facilitated the understanding of the epigenetic mechanisms underlying GC heterogeneity, offering an optimized decision-making and surveillance platform for GC patients.

**Supplementary Information:**

The online version contains supplementary material available at 10.1186/s13148-023-01478-w.

## Background

As a common malignant tumor with a poor prognosis, gastric cancer (GC) ranks fifth in morbidity and fourth in mortality among all cancers [1]. Despite the continuous innovation of precision medicine and individualized diagnosis and treatment of GC, the long-term survival rate of GC patients is still not optimistic [2]. Over the past few decades, the treatment of GC based on TNM staging and histological phenotype has significantly improved the survival of GC patients [3]. However, under the same treatment regimen, patients with the same histopathology type and stage tend to exhibit significantly different prognoses and treatment responses, indicating the limitations of current classification systems in addressing individual heterogeneity. The emergence of molecular classification based on gene expression holds promise for the individualized treatment of GC patients. Based on intrinsic genetic alterations, several classic molecular subtypes such as TCGA and Asian Cancer Research Group classifications were developed [4], demonstrating that based on GEPs, the dramatic potential of novel molecular classification is an effective tool for GC stratification.

Epigenetic alterations such as DNA methylation and miRNA regulation have been widely demonstrated to play critical roles in regulating gene expression and histological phenotypic variation [5, 6]. Aberrant DNA methylation as well as miRNAs had been shown to contribute to GC development, especially promoter hypermethylation and abnormal expression of miRNA, which could silence antioncogenes and thus contribute to tumorigenesis [7, 8]. Currently, molecular subtypes of GC based on DNA methylation regulation have been developed, and the potential of miRNA expression profiles for GC classification has been also demonstrated [9, 10]. However, it is still unclear whether epigenetic regulation of miRNAs and DNA methylation play a synergistic role in GC development, and if so, whether this role contributes to the classification of GC.

This study demonstrated the co-regulatory mechanism of miRNA expression and DNA methylation. Based on the integrated miRNA expression and DNA methylation profiles, we developed four molecular subtypes with significant differences in clinical traits and molecular features via an integrated clustering algorithm. Further validation in four independent multicenter cohorts demonstrated the stability of these four epigenetic-driven GC molecular subtypes. In addition, the correlations between our classifications and clinical traits, published classifications, epigenetic and genomic features, immune landscape and immunotherapy response, and potential therapeutics were also investigated.

## Materials and methods

### Data collection

**Fig. 1:**
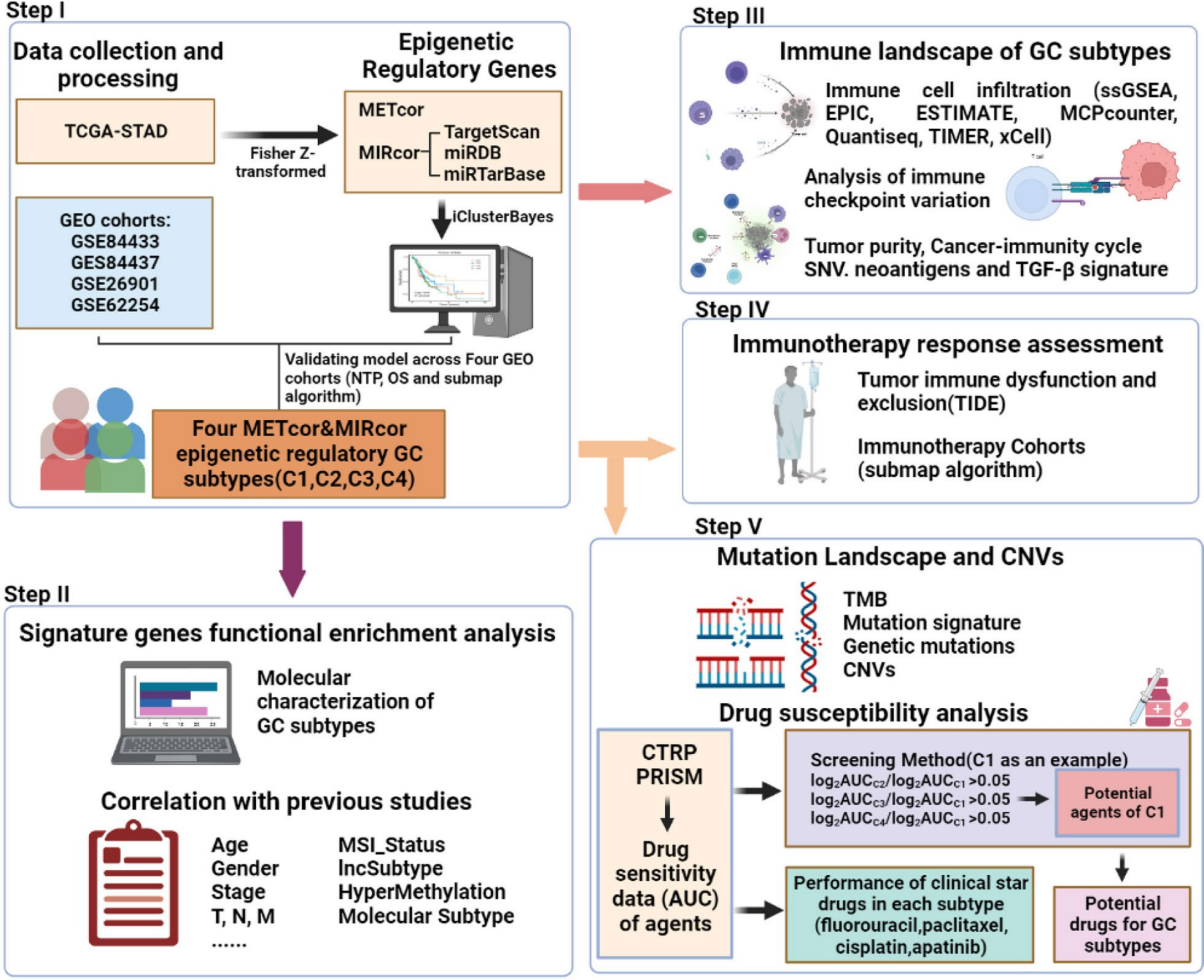
The flowchart of this study (Created with BioRender.com). The construction of molecular subtypes of GC driven by epigenetic regulation-related genes and the analysis of GC subtypes in terms of prognosis, functional analysis, clinical features, correlation with previously published subtypes, and further exploration of the immune landscape, immunotherapeutic potential, multi-omics alterations, and drug treatment differences of GC subtypes

Figure 1 summarizes the workflow of our study. A total of 1521 GC samples in five independent datasets, including GSE84433 (*n* = 357), GSE84437 (*n* = 433), GSE26901 (*n* = 109), GSE62254 (*n* = 300), and TCGA-STAD (*n* = 322), were systematically retrieved from the Gene Expression Omnibus (GEO, https://www.ncbi.nlm.nih.gov/) and TCGA (http://cancergenome.nih.gov/) databases, respectively. From the TCGA GDC database, DNA methylation (HumanMethylation 450), miRNA expression, and somatic mutation data were downloaded. Copy number variations (CNVs) were retrieved from FireBrowse (http://firebrowse.org/) using the Genomic Identification of Significant Targets in Cancer 2.0 (GISTIC2.0) algorithm [11]. Genes with deletion values above 20% were removed from the mRNA expression matrix and the normalized data of FPKM was further processed in the form of log2 (TPM + 1). Additional file 2: Table S1 contains the baseline data for the five cohorts. In addition, six immunotherapy cohorts including GSE67501, GSE100797, GSE136961, GSE111636, GSE140901, and GSE91061 were collected to predict the immune response capabilities of distinct GC subtypes.

### Screening and extraction of METcor and MIRcor genes

DNA methylation-correlated (METcor) and microRNA-correlated (MIRcor) genes were screened as follows: for METcor genes, based on 396,065 methylated CpG sites and 16,878 expressed mRNAs in TCGA-STAD dataset, 76,710 CpG sites were paired with 11,475 mRNAs, and a total of 76,710 CpG-mRNA pairs were obtained. For MIRcor genes, 938,564, 322,135, and 21,265,143 interacting miRNA-mRNA pairs in three databases including miRDB, miRTarBase, and TargetScan were screened, respectively [12–14]. 36,398 pairs of interacting miRNAs (n = 1871) and mRNAs (*n* = 6556) were further acquired after taking the intersection with the TCGA cohort.

1. Next, the Pearson correlation coefficients of CpG sites and miRNAs with the corresponding regulatory genes were calculated and the coefficients were further transformed by the Fisher Z-transformation method to stabilize the variance.
2. Although we did not observe a significant negative correlation phenomenon similar to CpG-mRNA among miRNA-mRNA pairs (Additional file 1: Fig. S1A), the negatively correlated miRNA-mRNA pairs were only retained for the following reasons: (i) generally, miRNAs negatively regulate downstream gene expression. In the process of cancer development and progression, microRNAs can promote cancer development by repressing the expression of tumor suppressor genes and halt the growth of cancer by inhibiting the translation of mRNAs produced by oncogenes [8]. (ii) In vitro experiments have revealed that miRNAs can promote gene expression [15], but this phenomenon has not been widely verified in vivo experiments. (iii) A majority of databases available for predicting miRNA-mRNA interactions have been created based on the fact that miRNAs may combine with genetic elements targeting mRNAs (usually in the 3! UTR) to regulate gene expression. Apparently, accurately predicting positive miRNA-mRNA pairs is presently challenging. Finally, with reference to previous studies, based on Fisher Z-transformed correlation coefficients with 95% confidence interval lower bounds, 803 METcor genes and 669 MIRcor genes were identified (< − 1.96, *P* < 0.05) [16].

### Identification of GC epigenetic regulatory subtypes and screening for signature genes

Based on 803 METcor and 669 MIRcor gene expression matrix, integrated clustering was performed using the iClusterBayes method to identify GC subtypes, which was implemented via the *iClusterPlus* R package. The optimal number of clusters driven by GC epigenetic genes was determined to be 4 according to Bayesian information criterion (BIC) and deviation ratio plot (Additional file 1: Fig. S1B). Subsequently, to screen the characteristic genes of distinct subtypes, with the help of the *limma* package, gene expression differential analysis was carried out [17]. Genes were arranged in descending order of log2 fold change. Considering that overfitting and underfitting may lead to the instability of GC subtypes, the top 500 differential genes of each subtype were selected as subtype characteristic genes.

### Validating the robustness of GC epigenetic subtypes

To evaluate the robustness of our epigenetic subtypes, nearest template prediction (NTP), an algorithm that predicts sample type based on characteristic genes was employed in four independent GEO cohorts (including GSE84433, GSE84437, GSE26901, GSE62254) to identify GC subtypes [18]. Besides, the subclass mapping (SubMap) algorithm was utilized to investigate the similarity of classifications in distinct gene expression profiles (GEPs) [19], further confirming the accuracy of our subtypes. Notably, both *P*-value and adjusted *P*-value < 0.05 were regarded as significant for subtypes similarity.

### Gene functional enrichment analysis

Functional enrichment analysis based on the *clusterProfiler* package was carried out to investigate the underlying biological mechanisms of the GC subtypes. The MSigDB database was used to source the gene sets used for annotation, which included 9997 gene sets (186 KEGG gene sets, 292 Biocarta gene sets, 7658 GO biological process gene sets, 196 PID gene sets, 1615 Reactome gene sets, and 50 cancer hallmark gene sets).

### Collection of published molecular subtypes of GC

To assess potential links between our epigenetic subtypes and traditional classifications, four conventional molecular subtypes of GC were systematically retrieved, including Chen et al. long-noncoding RNA (lncRNA)-based subtype (a method to classify GC by integrating tumor-specific lncRNA expression profiles, including three subtypes lncSubtype1, lncSubtype2, and lncSubtype3, which have different clinical and multi-omic features) [20], microsatellite status including microsatellite instability (MSI)- high (H), MSI- low (L), and microsatellite stability (MSS), CpG island methylation phenotype (CIMP) including CIMP-H, CIMP-Epstein–Barr Virus (EBV), colorectal cancer (CRC) CIMP-L, gastroesophageal adenocarcinomas (GEA) CIMP-L, and non-CIMP and molecular subtypes from TCGA study including chromosomal instability (CIN), EBV, genomic stability (GS), hypermutated single nucleotide variants (HM-SNV), and MSI [21, 22].

### Immune landscape of GC epigenetic subtypes

The single-sample gene-set enrichment analysis (ssGSEA) algorithm developed in the *GSVA* package was utilized to analyze the infiltration abundance of 28 immune cells to define the immunological landscape of the four subtypes [23]. In addition, to confirm the stability of the ssGSEA results, the remaining six immune cell infiltration assessment methods (including EPIC, ESTIMATE, MCPcounter, Quantiseq, TIMER, and xCell) were further performed [24]. Apart from that, to explore the expression differences of immune checkpoints (ICPs) for different subtypes, the expression of 27 ICPs including TNF family, B7 family, and other molecules for each sample were extracted from the TCGA cohort [25–27].

### Assessment of immunotherapy response in GC subtypes

To explore immunotherapy responses in patients with different subtypes, six immunotherapy cohorts including GSE67501, GSE100797, GSE136961, GSE111636, GSE140901, and GSE91061 were collected. SubMap algorithm was utilized to analyze the resemblance between the TCGA cohort and the GEO immunotherapy datasets to distinguish subtypes with a better immunotherapeutic response. In addition, some immune-related indicators were systematically collected and calculated, including tumor immune dysfunction and exclusion (TIDE) [28, 29], and cancer immunity cycle (CIC) [30].

### Mutation landscape and CNVs in each subtype

To explore differences in the multi-omics landscape between epigenetic subtypes, we further mined the representation of different subtypes in mutations and CNVs, as well as the associations between specific mutational signatures and our subtypes [31]. TMB was employed to assess DNA mutations per mega-byte (Mut/Mb) in GC via *Maftools* package. A waterfall plot of the top 20 most frequently mutated genes was visualized by *Maftools* and *ComplexHeatmap* packages. The top 20 amplified (AMP) and homozygous deletion (HOMDEL) chromosomal regions were visualized using the *ComplexHeatmap* package to reveal chromosomal alterations across different subtypes.

### Drug susceptibility analysis for each subtype

With reference to the study methodology of Yang et al. [32], we developed potentially sensitive drugs for each subtype. Drug susceptibility information for GC was downloaded from the Cancer Treatment Response Portal (CTRP) (https://portals.broadinstitute.org/ctrp.v2.1/) and PRISM (https://depmap.org/portal/) websites. Hematopoietic and lymphoid tissue cell lines were eliminated and 266 therapeutic agents in 658 cell lines from CTRP, as well as 1285 therapeutic agents in 474 cell lines from PRISM, were further obtained. Subsequently, based on the *pRRophetic* package, a ridge regression model was employed to evaluate the drug sensitivity for each sample, and the AUC (area under the dose–response curve) value was used as an indicator of the sensitivity. Greater drug sensitivity is reflected by a lower AUC value. K-nearest neighbor (k-NN) algorithm was conducted to fill in the missing values of the AUC matrix (drugs with more than 20% missing values were removed before processing).

Subtype-specific drugs were screened based on the following criteria: the average AUC of each subtype of compounds was defined as a (corresponding to C1), b (corresponding to C2), c (corresponding to C3), and d (corresponding to C4), respectively, and when log2 (b/a), log2 (c/a), and log2 (d/a) were all > 0.05, this drug was considered as a potential drug for C1. Furthermore, some chemotherapy and targeted therapy drugs commonly used in clinical practice including 5-fluorouracil (5-FU), cisplatin, paclitaxel, and apatinib were collected to investigate the variations in sensitivity of distinct subtypes.

### Statistical analysis

In this study, the statistical analyses were completed based on R software (version 4.1.1). Pearson’s Chi-square test or Fisher’s exact test was employed to analyze categorical variables, while the Kruskal–Wallis rank-sum test or t test was performed to evaluate the variations between continuous variables and prior to testing, the Shapiro–Wilk method was utilized to check the normality of the variables. The survival differences among subtypes were assessed by Kaplan–Meier analysis with the log-rank test and multivariate Cox regression analyses in the *survival* and *survminer* packages. Pearson correlation was used to assess the strength of the linear relationship between two variables. A two-tailed *P* < 0.05 was regarded as statistically significant.

## Results

### Identification and comprehensive analysis of METcor and MIRcor genes

**Fig. 2:**
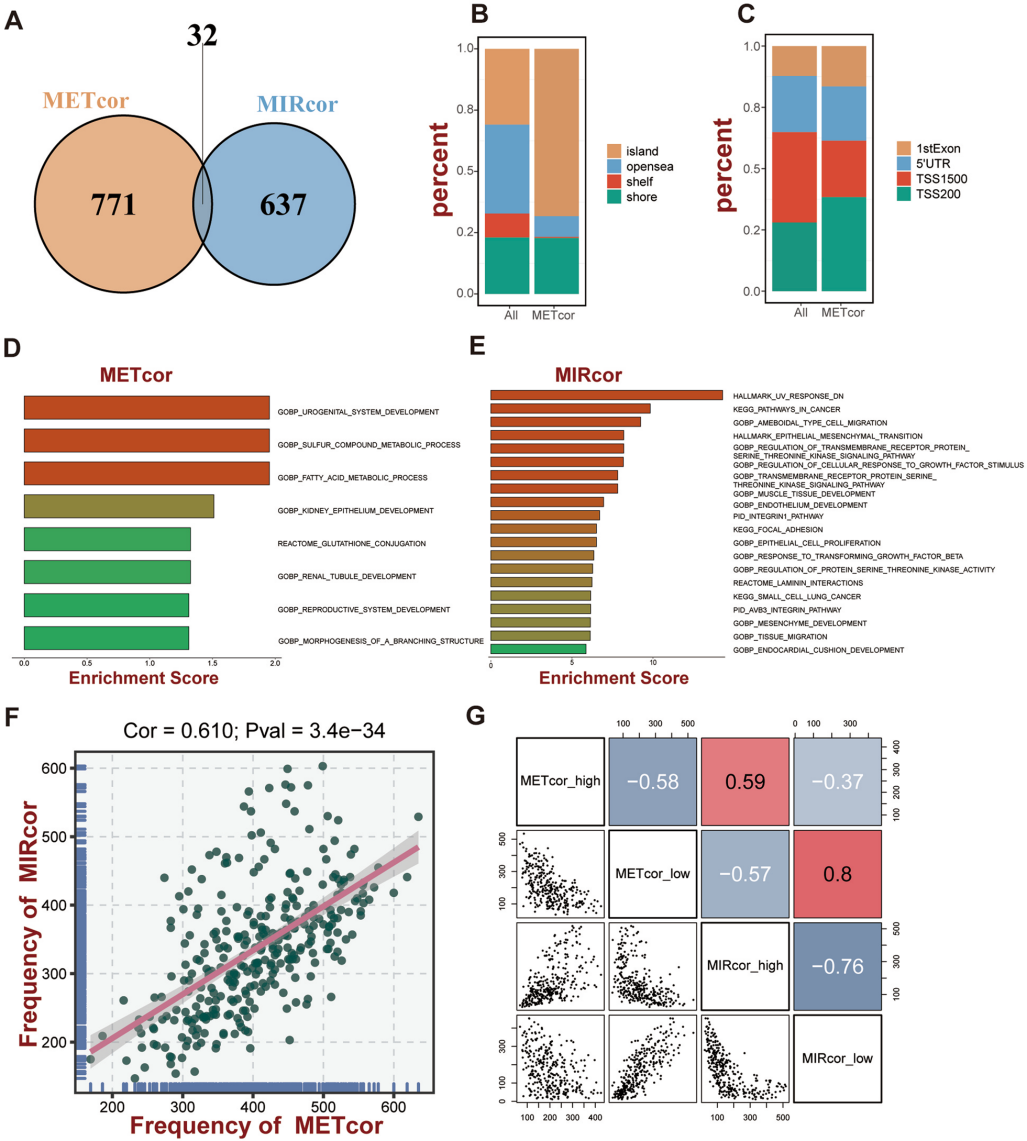
Screening of METcor and MIRcor genes in TCGA-STAD. **A** Overlap of the METcor and MIRcor genes. **B**, **C** Proportional frequency of promoter CpG sites based on their distance relative to CpG islands and genomic positions, respectively. Shore, 0–2 kb upstream or downstream from CpG island; Shelf, 2–4 kbp upstream or downstream from CpG island; Opensea, other regions of the genome. **D**, **E** Functional enrichment analysis of the METcor and MIRcor genes. **F** Correlation between the frequencies of aberrant METcor and MIRcor genes in each sample of the TCGA dataset. **G** Pairwise correlations among the frequencies of METcor_high, METcor_low, MIRcor_high and MIRcor_low genes, respectively

803 METcor (Additional file 2: Table S2) and 669 MIRcor genes (Additional file 2: Table S3) were screened out according to the above steps. Notably, only 32 genes (Additional file 2: Table S4) overlapped in METcor and MIRcor genes (Fig. 2A), suggesting unique mechanisms of DNA methylation and miRNAs in regulating gene expression. In addition, DNA methylation occurred more frequently in islands and transcription start site 200 (TSS200) compared to all probes, suggesting that it is more probable that methylation in these regions will contribute to the regulation of mRNAs (Fig. 2B, C). Functional enrichment analysis revealed that METcor genes were significantly involved in organ development, metabolism of sulfur-containing compounds and fatty acids, and glutathione conjugation reactions (*P* < 0.05, Fig. 2D). While MIRcor genes were overrepresented in cancer-related pathways, epithelial–mesenchymal transition, cell proliferation–adhesion–migration, and tissue development (*P* < 0.05, Fig. 2E). The distinct functional enrichment results revealed that they were involved in regulating dramatically different biological processes.

To investigate the correlation between the METcor and MIRcor genes aberrations, we ranked the expression of METcor and MIRcor genes in each sample from low to high, with genes located in the top 25% defined as low expression and those located in the bottom 25% defined as high expression. Notably, frequently abnormal MIRcor gene patients tend to exhibit frequent aberration of the METcor genes. (Cor = 0.610, *P* < 0.001, Fig. 2F). Meanwhile, in the pairwise comparison of METcor and MIRcor genes, it was found that there was a substantial correlation between the expressions of the METcor and MIRcor genes at high and low levels (Fig. 2G), implying that DNA methylation and miRNA expressions co-regulated the expressions of downstream genes in GC.

### Integrative clustering identified four GC subtypes

**Fig. 3:**
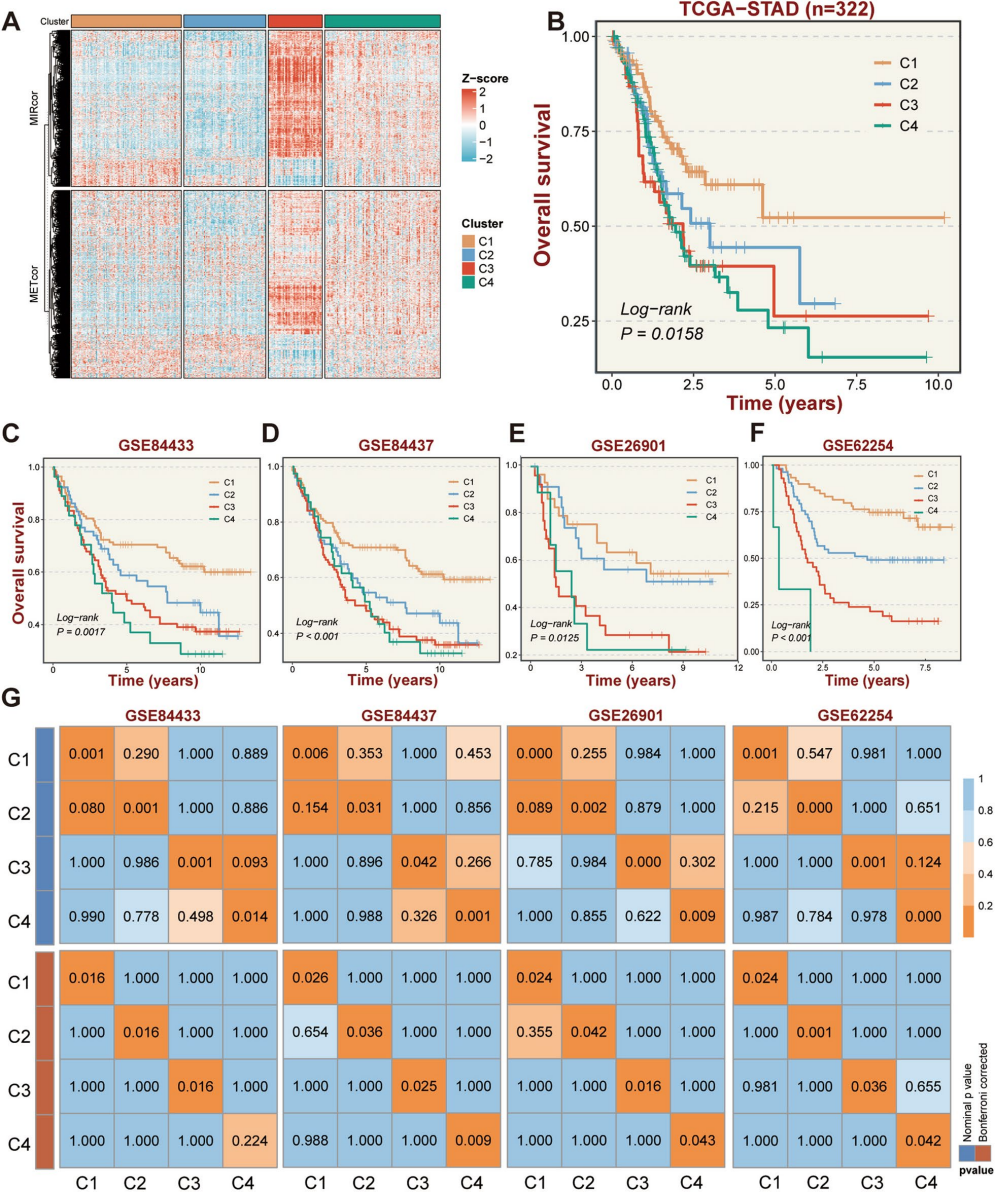
Identification and validation of GC subtypes by integrated clustering using METcor and MIRcor genes. **A** Heatmap shows the expression patterns of METcor and MIRcor genes of GC subtypes identified by integrated clustering. **B** Kaplan–Meier (K–M) survival curves for the GC subtypes of the TCGA cohort are shown for overall survival (OS). **C**–**F** K–M survival curves for the GC subtypes of the GEO cohorts (GSE84433, GSE84437, GSE26901, GSE62254) are shown for OS, respectively. **G** SubMap analysis reveals a significant correlation of gene expression profiles between GC subtypes of TCGA and GEO datasets

Under the premise of verifying the cooperative regulation of METcor and MIRcor genes, we performed the iClusterBayes algorithm to integrate METcor and MIRcor GEPs for cluster analysis. Ultimately, four subtypes were identified with cluster 3 (C3, *n* = 48) and cluster 4 (C4, *n* = 103) displaying higher METcor and MIRcor gene expression preferences compared to cluster 1 (C1, *n* = 98) and cluster 2 (C2, *n* = 73) (Fig. 3A). In addition, among four subtypes, C4 showed the worst prognostic outcome while C1 was the best (*P* = 0.0158, Fig. 3B). Subsequently, we explored the different expression patterns of METcor and MIRcor genes in distinct subtypes and found that in terms of frequency of occurrence, they appeared with the highest frequency in C3 subtype, while C4 displayed the lowest overall frequency among the GC subtypes (*P* < 0.05, Additional file 1: Fig. S1C). The frequencies of METcor_high and MIRcor_high genes in subtype C3 were the highest while the frequencies of METcor_low and MIRcor_low genes were the lowest among the four subtypes. (*P* < 0.0001, Additional file 1: Fig. S1D, E).

### Comprehensive validation of GC subtypes

To assess the accuracy and robustness of our classifications, the NTP algorithm was employed to forecast the clusters of GC samples in four independent GEO cohorts based on the feature genes of each subtype which were summarized in Additional file 2: Table S5. Highly similar to the categorization results of the TCGA-STAD cohort, all cohorts were precisely classified into four subtypes (Additional file 1: Fig. S2A–D). Consistently, survival differences among GC subtypes were verified in four GEO cohorts, with C4 performing the worst prognosis. (Fig. 3C–F). SubMap analysis displayed that the subtypes in four GEO cohorts were in significant concordance with those in the TCGA-STAD cohort (Fig. 3G). These aforementioned findings validated the stable performance of our epigenetically driven GC subtypes.

### Molecular characteristics of GC subtypes

**Fig. 4:**
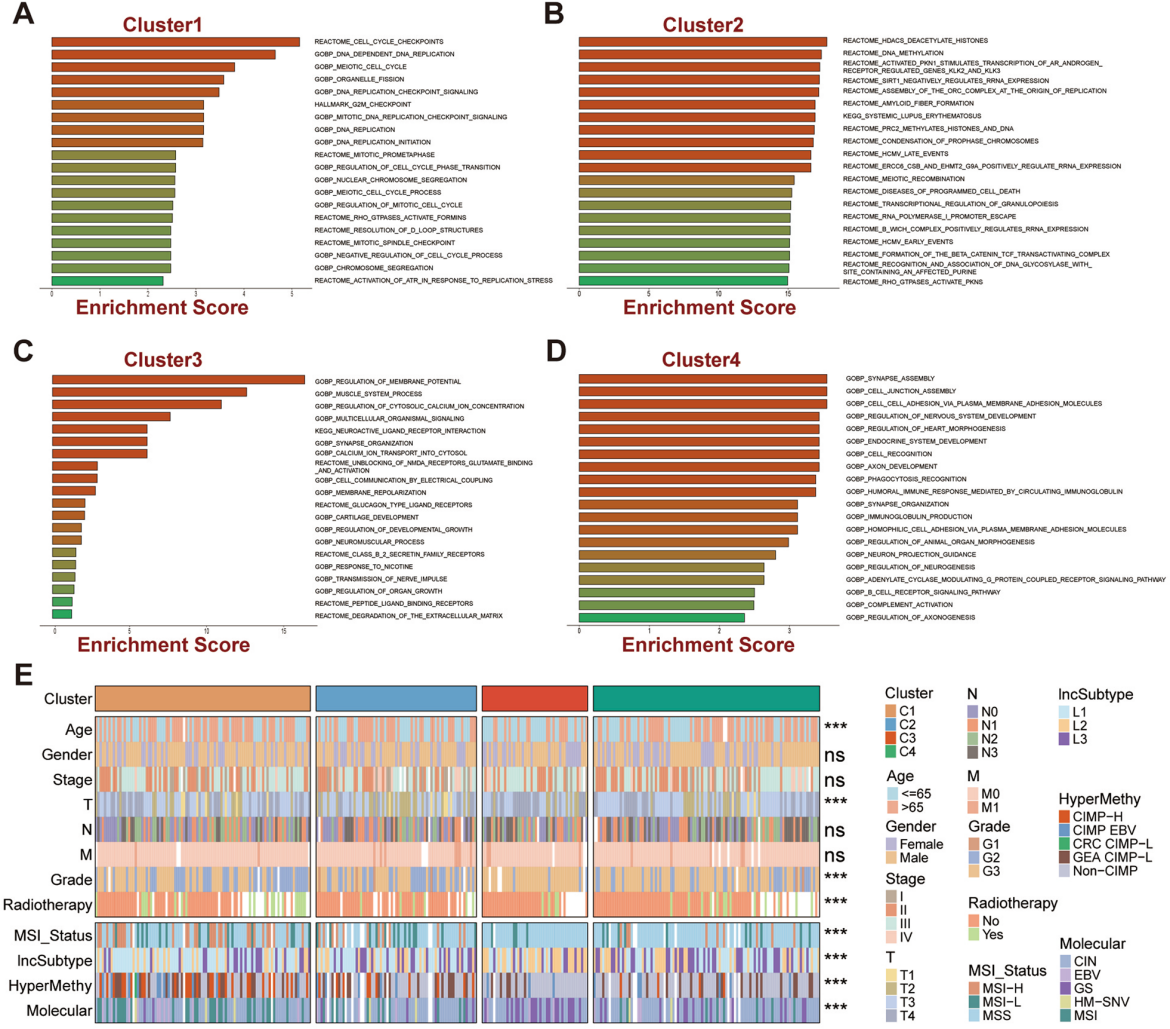
Molecular and clinical characterization of GC subtypes in TCGA dataset. **A**–**D** Functional enrichment analysis based on 2000 subtype-specific genes (500 specific genes per subtype). **E** Correlations of GC subtypes with clinical features and previous GC classifications. White denotes missing values. ‘ns’ represents no significance, ^*^P < 0.05, ^**^P < 0.01, ^***^P < 0.001,  ^****^P < 0.0001

To decipher the distinct biological characterization of GC subtypes, enrichment analysis was performed in TCGA-STAD cohort and revealed remarkable functional differences among the four molecular subtypes (*P* < 0.05, Fig. 4A–D). Specifically, C1 was mainly involved in DNA replication, cell cycle regulation, and cell division, which suggested that C1 might possess a tight association with cell proliferation and differentiation (Fig. 4A). Histone and DNA modifications and activated PKN1-stimulated processes such as transcription of the AR (androgen receptor) regulatory genes KLK2 and KLK3 were overrepresented in C2 (Fig. 4B), which exhibited active intracellular signaling. The C3 subtype was more involved in ligand–receptor binding and formation-related pathways (Fig. 4C), while the C4 subtype was significantly associated with immune-related functional pathways (Fig. 4D).

### Correlation between GC subtypes and clinical features

Further, the commonly shared clinical features of the GC subtypes were compared. It was observed that C3 and C4 were associated with advanced stage (T3 and T4, *P* < 0.001) and grade (G3, *P* < 0.001), validating the poorer prognostic outcome of C3/C4 patients. Subtype C1 possesses the largest number of older individuals (age > 65). Besides, significant correlations were observed among the GC subtypes and the four published subtypes. Specifically, C1 and C2 that performed a better prognosis were associated with MSI-H. C3 was remarkably related to MSS, Chen et al.’s lncSubtype2 (associated with diffuse-type and genomic stability GC with a moderate prognosis), and genomic stability (GS) subtype. C4 was correlated with MSS, Chen et al.’s lncSubtype3, chromosomal-instable (CIN) tumors, and non-CIMP (*P* < 0.001, Fig. 4E). Interestingly, the worst prognosis lncSubtype3 was characterized by pervasive TP53 mutations, chromatin instability, hypomethylation, and over-expression of oncogenic lncRNAs, which is strikingly similar to the performance of C4. These observations matched the distinctive aggression characteristics of our classifications.

### The immune landscape of four GC subtypes

**Fig. 5:**
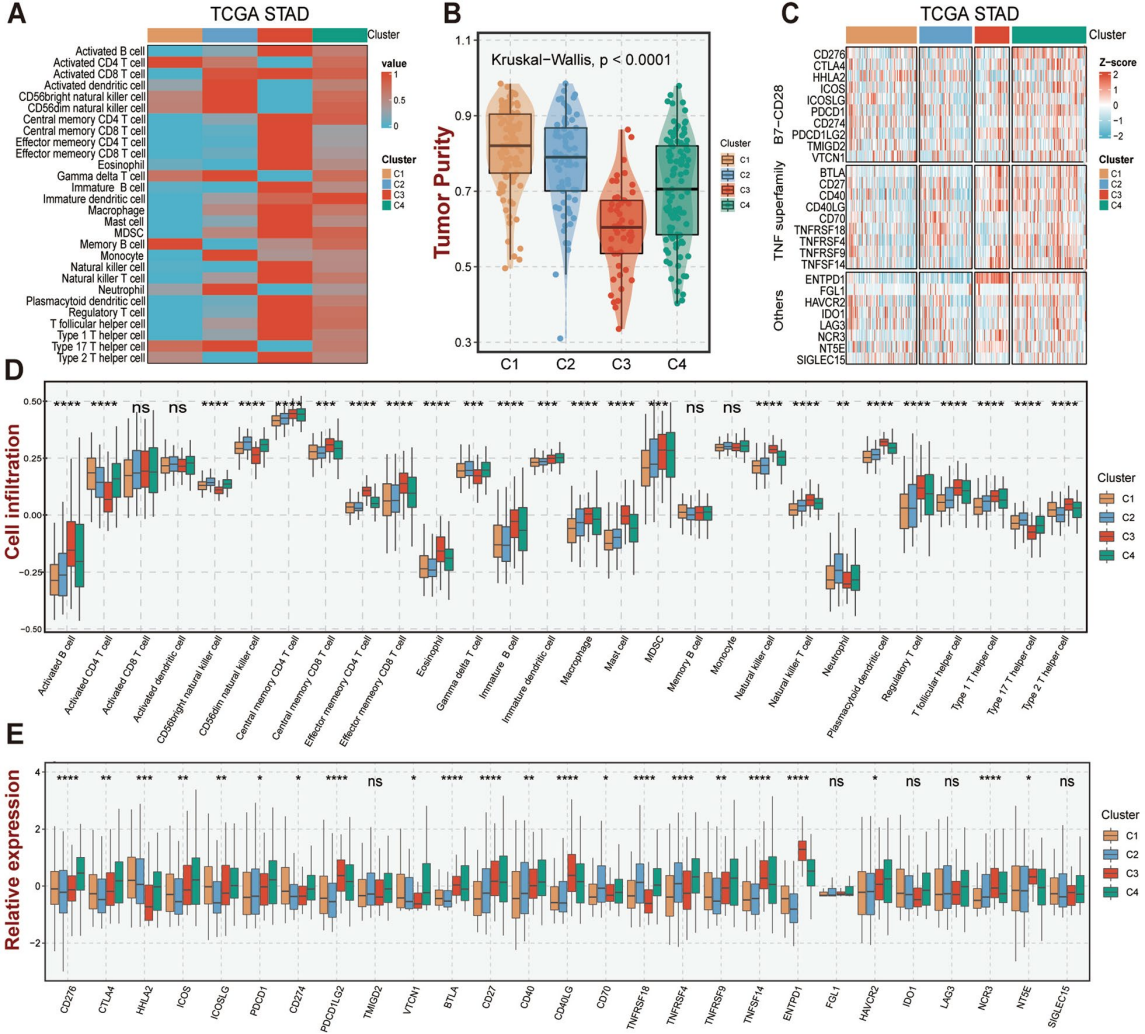
The immune landscape of GC subtypes in TCGA cohort. **A** Mean infiltration abundance of 28 immune cell types in GC subtypes. **B** Tumor purity of GC subtypes. **C** Heatmap of expression profiles for 27 immune checkpoint-related genes of GC subtypes. **D** Boxplot between GC subtypes and 28 immune cells infiltration abundance. **E** Boxplot of 27 immune checkpoint profiles for GC subtypes. ‘ns’ represents no significance, ^*^P < 0.05, ^**^P < 0.01, ^***^P < 0.001,  ^****^P < 0.0001

A deeper investigation of the immunological landscape of four GC subtypes was conducted in order to understand the underlying relationships between our subtypes and immunity. The infiltration abundance of 28 immune cells in distinct GC subtypes was evaluated via the ssGSEA algorithm. Particularly, both C3 and C4 patients displayed a richer level of immune cell infiltration and higher immune scores (Fig. 5A, D; Additional file 1: Fig. S3A), whereas C3 exhibited a lower tumor purity and the highest stromal score (Fig. 5B; Additional file 1: Fig. S3B). In parallel, according to ICP comparative analysis results, the majority of the ICPs were noticeably up-regulated in C3 and C4 subtypes (Fig. 5C). However, the expression of several immune-related co-suppressor genes including *PDCD1LG2*, *BTLA*, *TNFRSF14*, *ENTPD1*, and *NTE5* were significantly higher in C3 while *CTLA-4*, *PDCD1*, and *HAVCR2* were higher in C4, whereas several immune-related co-stimulatory genes (*HHLA2*, *ICOS*, *ICOSLG*, *CD274*, *CD70*, *CD40*, *TNFRSF18*, *TNFRSF4*, and *TNFRSF9*) were dramatically up-regulated in C4 [11, 33] (*P* < 0.05, Fig. 5E).

**Fig. 6:**
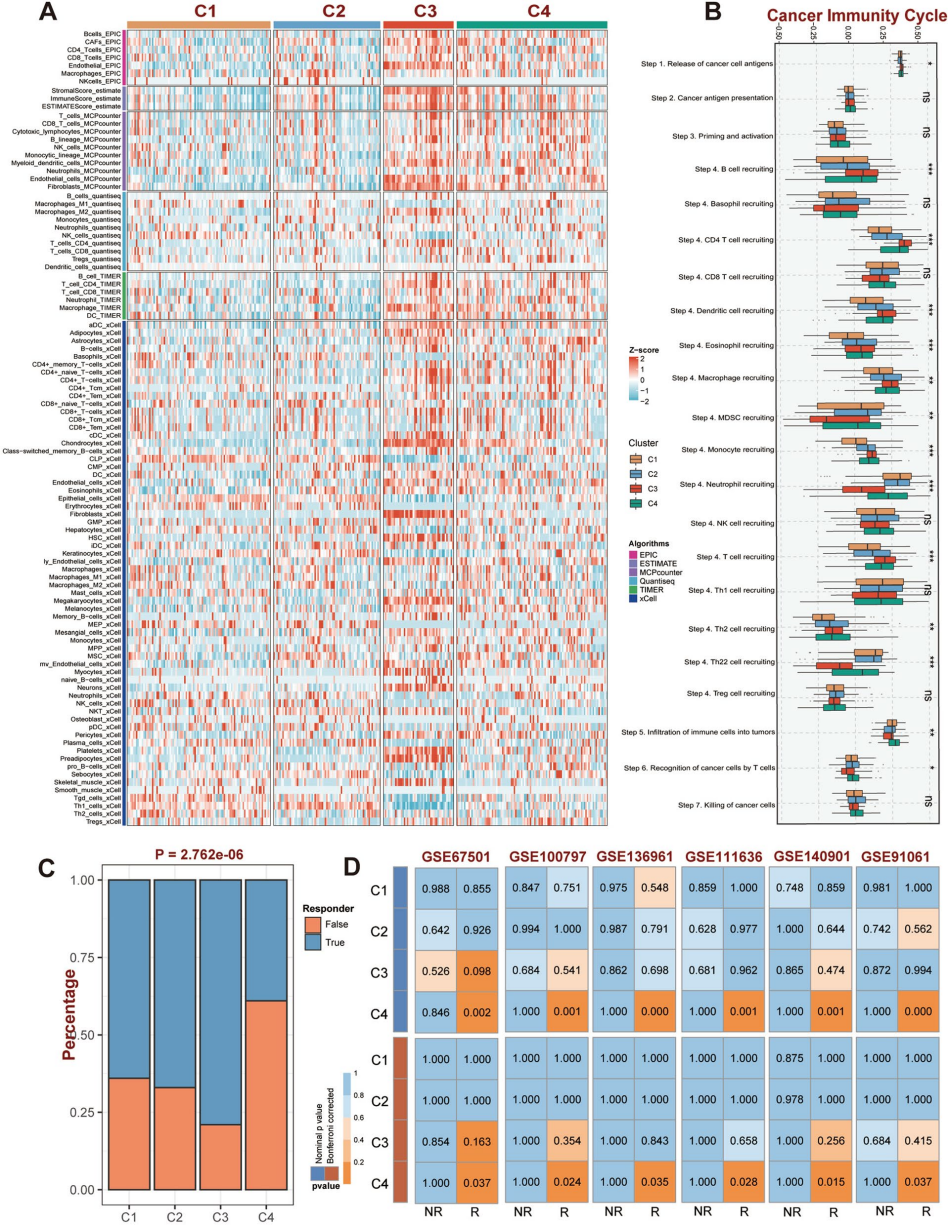
The further decipherment of immune landscape for GC subtypes of the TCGA dataset. **A** Heatmap of the remaining six immune cell infiltration assessment algorithms (EPIC, ESTIMATE, MCPcounter, Quantiseq, TIMER, and xCell) for our subtypes. **B** The performances of GC subtypes during the cancer immunity cycle. **C** The performance of GC subtypes in tumor immune dysfunction and exclusion (TIDE) of the TCGA cohort. **D** SubMap analysis reveals the similarity of GEPs between GC subtypes of TCGA and 6 GEO immunotherapy cohorts. ‘*R*’ represents responders, and ‘NR’ represents non-responders. ‘ns’ represents no significance, ^*^P < 0.05, ^**^P < 0.01, ^***^P < 0.001,  ^****^P < 0.0001

Notably, to ensure the specific analytical algorithms did not bias the results, six additional immune infiltration assessment algorithms, including EPIC, ESTIMATE, MCPcounter, Quantiseq, TIMER, and xCell, were conducted to confirm the accuracy of the above results. As expected, similar results were observed with the C3 and C4 subtypes exhibiting higher infiltration abundance of immune cells (Fig. 6A). In addition, comparative analysis demonstrated significant differences among the four subtypes in distinct CIC steps. Specifically, C3 exhibited negative performance in both immune cell infiltration into the tumor and T cell recognition of cancer cells, indicating that patients with the C3 subtype are likely to be in T cell exhaustion (*P* < 0.05, Fig. 6B).

According to the research exists, immune evasion and immunological activation were likely to be intimately associated with the C3 and C4 subtypes, respectively. For further verification, the single nucleotide variants (SNV) derived neoantigens scores and pan-fibroblast transforming growth factor b (TGF-β) response signature scores were calculated. In general, higher SNV neoantigen scores indicated greater immunogenicity, while higher TGF-β scores revealed greater immunotherapy resistance and immune evasion mediated by the TGF pathway [34–36]. Consistently, C3 exhibited the lowest SNV-derived neoantigens score (*P* < 0.05, Additional file 1: Fig. S3C), and the highest TGF-β response signature score (*P* < 0.001, Additional file 1: Fig. S3D), validating the immunosuppressive and immune evasion properties of C3 patients.

### Immunotherapy response assessment of four GC subtypes

Taking into account the significant differences in the immunological landscape in four epigenetic subtypes, several immune-related indicators were systematically collected and calculated. TIDE analysis suggested that patients in C4 subtype exhibited a higher immune response rate than other subtypes (*P* < 0.001, Fig. 6C). Furthermore, based on SubMap algorithm, we assessed the resemblance of GEPs among the identified GC subtypes and six immunotherapy cohorts, the results indicating that patients with subtype C4 all exhibited better immune responses across six immunotherapy cohorts (Fig. 6D).

### Correlation of GC subtypes with mutations landscape and CNVs

**Fig. 7:**
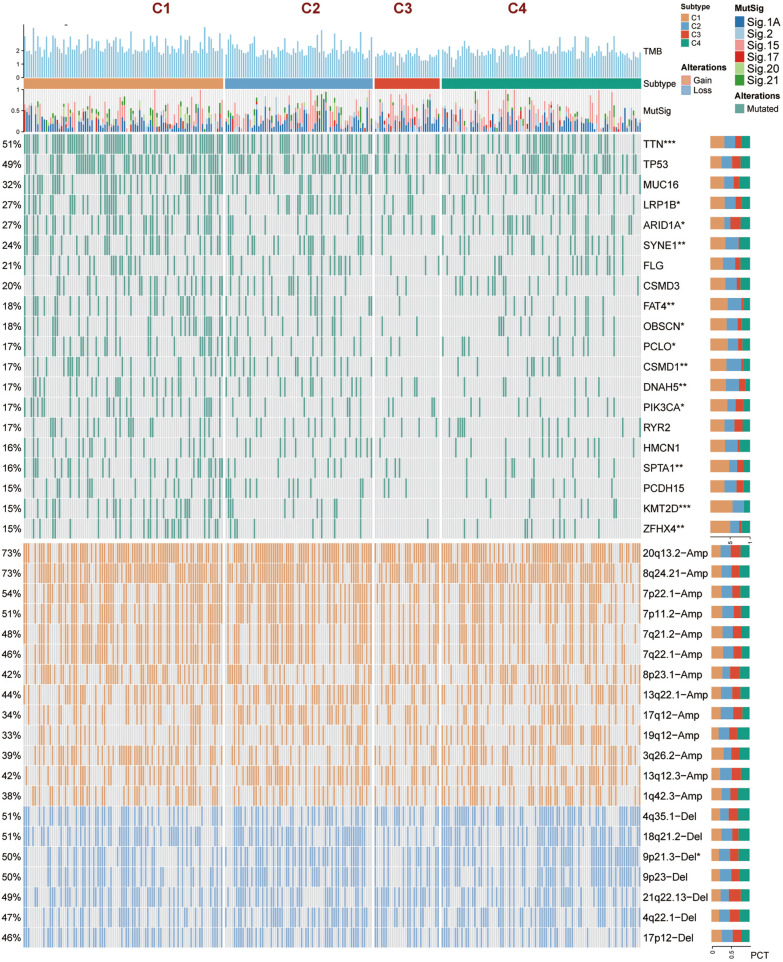
Multi-omics alterations of GC subtypes in TCGA cohort. The somatic mutational landscape of the top 20 frequently mutated genes and the CNVs landscape of the top 20 AMP and Homdel chromosome fragments for GC subtypes. The proportion of these variants in the GC subtypes is denoted on the right. ^*^P < 0.05, ^**^P < 0.01, ^***^P < 0.001, ^****^P < 0.0001

To decipher the genomic alterations in GC subtypes, the characteristic mutations and CNVs of the four subtypes were further explored. Specifically, the mutation frequency of *TTN, ARID1A*, *FAT4*, *PIK3CA*, *SPTA1*, *KMT2D*, and *ZFHX4* was significantly higher in C1 subtype; *CSMD1* performed higher mutation frequencies in C2 subtype. In parallel, the deletion frequency of 9p21.3 was dramatically higher in C4 subtype (Fig. 7). Moreover, mutation signature analysis displayed that signature 1A and signature 15 accounted for a greater percentage than the other GC-related signatures, offering fresh information for a more thorough investigation of the pathogenic mechanism of GC [31].

### Potential therapeutic agents for specific subtypes

**Fig. 8:**
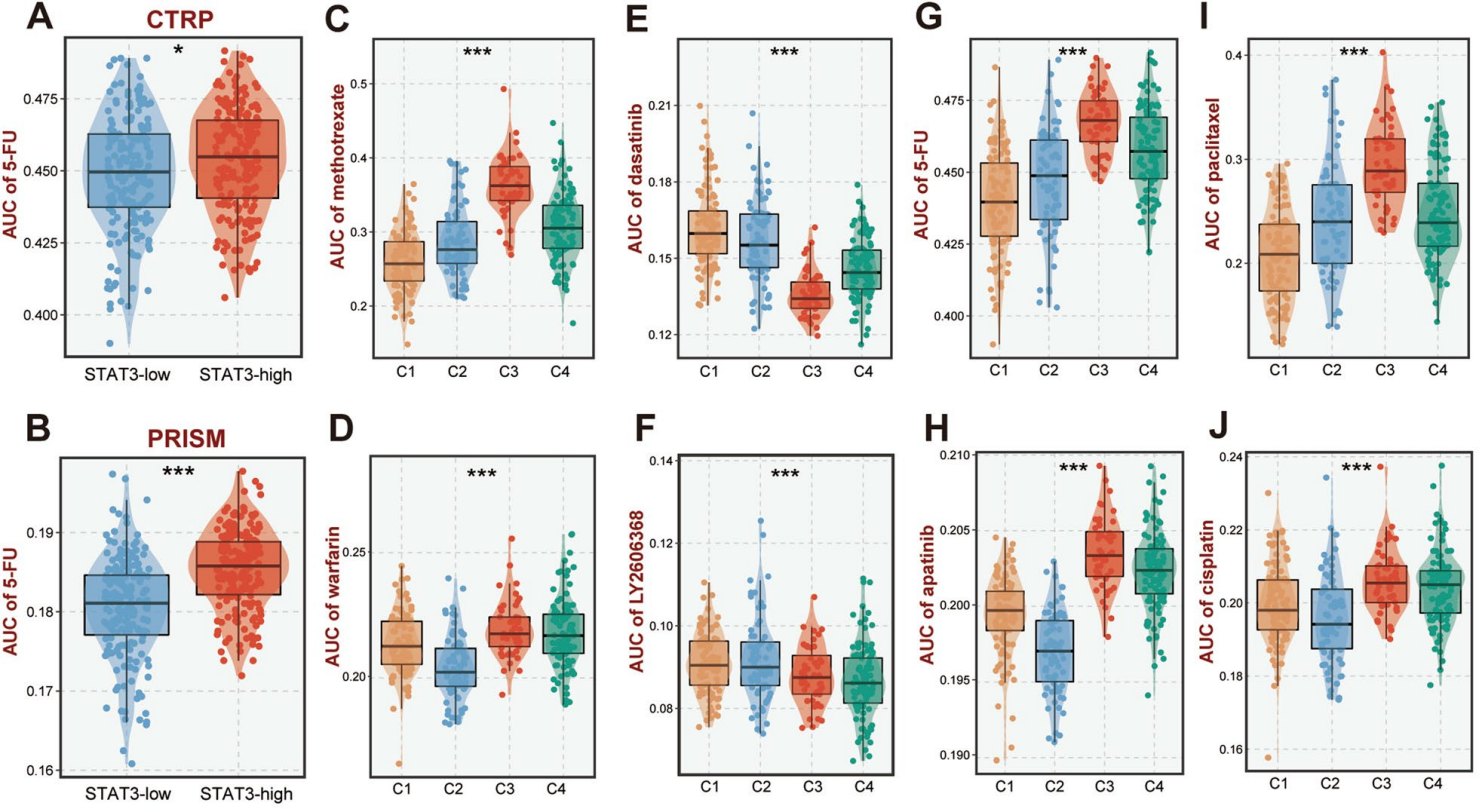
Potential therapeutic agents for each subtype. **A**, **B** Consistency and robustness of two prediction models (CTRP and PRISM), the low expression of STAT3 correlates with sensitivity to 5-fluorouracil (5-FU). **C**–**F** The most probable potential therapeutic agents for each subtype. **G**–**J** Susceptibility prediction of GC subtypes based on CTRP and PRISM for 4 first-line drugs (5-FU, apatinib, paclitaxel, and cisplatin). ^*^P < 0.05, ^**^P < 0.01, ^***^P < 0.001, ^****^P < 0.0001

Differential drug response analysis was conducted to find agents with the lowest AUCs in comparison with the other three subtypes to find prospective treatment agents for certain GC subtypes. The widely used GC chemotherapy chemical 5-fluorouracil was utilized to investigate if the projected drug sensitivity was consistent with its clinical efficacy to ensure that the drug sensitivity information obtained was reliable. Studies have demonstrated that decreased *STAT3* expression or malfunction forecasts increased 5-FU sensitivity in GC [37]. Thus, depending on the median levels of *STAT3* expression, the TCGA cohort of patients was split into high- and low-*STAT3* expression subgroups. In line with the published research, patients with reduced *STAT3* expression exhibited lower 5-FU AUCs in the CTRP and PRISM databases. (*P* < 0.05, Fig. 8A, B), illustrating the significant accuracy of the predicted compounds reaction. Subsequently, the potential therapeutic drugs for specific subtypes were screened, respectively. Specifically, patients of C1 were sensitive to methotrexate; C2 was probably sensitive to warfarin; the sensitivity of dasatinib was significantly higher in C3 subtype; and LY2606368 (Prexasertib) probably be a potential therapeutic agent for C4 GC patients (*P* < 0.001, Fig. 8C–F).

According to the current practice guidelines for the treatment of GC, we further explored the sensitivity of commonly used drugs including 5-fluorouracil, paclitaxel, apatinib, and cisplatin in patients with different subtypes [38]. In particular, C1 subtype was sensitive to 5-FU and paclitaxel, while C2 patients benefit more from apatinib and cisplatin (*P* < 0.001, Fig. 8G–J). Notably, C3 displayed the highest AUCs to these drugs, indicating that C3 subtype tends to be resistant to chemotherapy and targeted therapy.

## Discussion

Transcriptomic dysregulation mediated by epigenetic mechanisms is critical in the uneven evolution of GC. Currently, molecular subtypes of GC based on DNA methylation regulation have been developed, and the classification potential of miRNA expression profiles has been widely confirmed [9, 10]. However, it is still unclear whether epigenetic regulation of miRNAs and DNA methylation play a synergistic role in GC advancement, and if so, whether it contributes to the classification of GC. Here, we found that the aberrant frequencies of MIRcor genes and METcor genes are significantly co-regulated. Depending on the integrated miRNA expression and DNA methylation profiles, four molecular subtypes with significant differences in clinical traits and molecular features were developed and validated in 1521 GC samples from five independent cohorts. The correlations between our subtypes and clinical traits, published subtypes, epigenetic and genomic features, immune landscape and immunotherapy response, and potential therapeutics were further investigated.

In this study, patients of C1 subtype exhibited better survival, patients with subtype C4 showed the worst prognosis, and the prognosis of C2 and C3 subtypes lay somewhere in between. This was consistent with our findings that C4 subtype tends to perform advanced tumor stage and higher grade. Pathway enrichment analysis suggested that these four subtypes were endowed with cell proliferation and transformation, intracellular signaling, ligand–receptor formation, and immune-related pathways, respectively, illustrating the significant differences in biological functions of the four epigenetic subtypes. Moreover, potential links between the four subtypes and published subtypes were further investigated to characterize the underlying properties of distinct epigenetic GC subtypes. It was observed that C1 subtype is significantly associated with MSI-H subtype, which has been widely shown to perform better prognosis [39–41], whereas the C4 subtype is strongly related to MSS and lncSubtype3 tumors, further validating its dismal prognostic outcomes [20, 42]. In addition, the similar performance of C4 subtype with lncSubtype3 seems to guide us to investigate the relationship between lncRNA and epigenetic alterations in GC in more depth. Notably, our findings indicated that patients of C1 subtype typically exhibit higher tumor purity. As previous reports, a higher tumor purity generally reveals a better prognosis in GC and more likely to benefit from adjuvant chemotherapy [43, 44], which not only validated the better survival of C1 subtype but also suggested that C1 patients might benefit more from neoadjuvant chemotherapy.

Considering the high correlation between C4 subtype and tumor immunity, the immune landscape of four subtypes was further investigated. Although C3 and C4 subtypes exhibited a significantly elevated abundance of immune cell infiltration, distinct immunological properties were observed in these two subtypes, respectively. Specifically, the expression of several immune-related co-suppressor genes including *CTLA-4*, *PDCD1*, *PDCD1LG2*, *BTLA*, *TNFRSF14*, *ENTPD1*, *HAVCR2*, and *NTE5* were significantly higher in C3, whereas several immune-related co-stimulatory genes (*HHLA2*, *ICOS*, *ICOSLG*, *CD274*, *CD70*, *CD40*, *TNFRSF18*, *TNFRSF4*, and *TNFRSF9*) were dramatically up-regulated in C4, suggesting that the C3 may be an immunosuppressive subtype, while the C4 tends to be an immune-activating subtype. Alternatively, the C3 subtype displayed the lowest scores in tumor immune steps such as immune cell infiltration into the tumor and T cell recognition of cancer cells, suggesting that patients with the C3 subtype may be in a state of T cell exhaustion, further leading to immunosuppression and immune evasion. Besides, C3 performed the lowest SNV-derived neoantigens score and the highest TGF-β response signature score, validating the immune evasion and resistance to immunotherapy properties of C3 patients [34, 35, 45]. Notably, TIDE analysis displayed that patients with C4 subtype exhibited the highest sensitivity to immunotherapy, which was further validated in six independent immunotherapy cohorts based on the SubMap algorithm, suggesting that patients with C4 subtype are more probably to benefit from immunotherapy.

Subsequently, we investigated the mutational landscape of GC subtypes to characterize the somatic mutations and CNVs that may drive GC subtypes. The mutation frequencies of *TTN, PIK3CA*, and *KMT2D* were significantly higher in C1 subtype. High mutation frequency of *TTN* tends to predict better survival outcomes, while mutations of *PIK3CA* were associated with a better prognosis in older individuals in GC [46, 47], which validated the results of the survival analysis and clinical traits comparisons. In addition, the study by Li et al. showed that mutations in *KMT2D* are closely related to the proliferation of GC [48], which is consistent with the cell proliferation and transformation properties of the C1 subtype in the functional analysis. Moreover, among the top 20 frequently mutated genes in GC, most genes showed the highest mutation frequency in C1 subtype, suggesting that C1 subtype is likely to be a mutation-driven subtype.

Based on multiple pharmacological databases and comprehensive bioinformatics algorithms, four potential agents sensitive to C1, C2, C3, and C4 were obtained, respectively. 5-FU, cisplatin, paclitaxel, and apatinib are currently the mainstay of combination chemotherapy or targeted therapy for GC patients [38]. Our results indicated that C3 subtype displayed the lowest sensitivity to all four drugs, suggesting that chemotherapy and targeted treatment may be less effective for those with C3. On the other hand, patients with C1 displayed a higher sensitivity to 5-FU and paclitaxel, while C2 patients benefit more from apatinib and cisplatin, providing new insights into the individualized therapy for distinct GC subtypes.

Although our classification is a promising comprehensive platform to stratify GC patients, several restrictions should be recognized. Firstly, since each sample used in our investigation was retrospective, future validation of GC subtypes should be carried out in prospective fresh samples. Secondly, due to limited resources and capacity, we did not explore the link between epigenetic alterations in GC and intrinsic genetic alterations. Therefore, the study of epigenetic regulatory mechanisms of GC still needs to be explored in greater depth.

## Conclusions

Based on the epigenetically regulated GEPs, we developed four robust GC molecular subtypes, which not only facilitated the comprehension of the epigenetic mechanisms involved in GC heterogeneity but also offered a viable platform for enhancing decision-making and surveillance procedure for specific GC patients.

## Supplementary Information

**Additional file1**. **Figure S1**: Differential expression of METcor and MIRcor genes in TCGA dataset. (A) Distribution of CpG-mRNAs and miRNA-mRNA correlation coefficients in the TCGA cohort. (B) The optimal number of clusters is 4 (k+1) determined by the Bayesian information criteria (BIC) and deviance ratio plot between 2 and 5 clusters. (C) Differential analysis of METcor and MIRcor gene expression frequencies in GC subtypes. (D) Expression frequencies of METcor_high(highly expressed), METcor_low(lowly expressed) and METcor_all genes in GC subtype. (E) Expression frequencies of MIRcor_high, MIRcor_low and MIRcor_all genes in GC subtypes. ∗P < 0.05, ∗∗P < 0.01, ∗∗∗P < 0.001, ∗∗∗∗P < 0.0001. **Figure S2**: The METcor and MIRcor gene expression patterns of the TCGA cohort and the GEO cohorts are consistent. (A–D) The nearest template prediction (NTP) in GEO cohorts (GSE84433, GSE84437, GSE26901, GSE62254) via 2000 subtype-specific genes was in excellent agreement with the TCGA cohort. **Figure S3**: Several indicators reflect the tumor immune microenvironment of GC subtypes in TCGA cohort. (A–B) The immune and stromal scores of GC subtypes. (C) The single nucleotide variants (SNV) derived neoantigens scores of GC subtypes. (D) Differential analysis of pan-fibroblast transforming growth factor b (TGF-β) response signature scores. ∗P < 0.05, ∗∗P < 0.01, ∗∗∗P < 0.001.

**Additional file2**. **Table S1**: Clinical characteristics of the TCGA-STAD and GEO cohorts. **Table S2**: List of METcor genes. **Table S3**: List of MIRcor genes. **Table S4**: List of 32 overlapping METcor and MIRcor genes. **Table S5**: Subtype-specific genes in this study.

## Data Availability

Public data used in this work can be acquired from the TCGA Research Network portal (https://portal.gdc.cancer.gov/) and Gene Expression Omnibus (GEO, http://www.ncbi.nlm.nih.gov/geo/).

## Abbreviations

GC: Gastric cancer
GEPs: Gene expression profiles
GEO: Gene Expression Omnibus
GS: Genomic stability
GSEA: Gene-set enrichment analysis
METcor: DNA methylation-correlated
MIRcor: MicroRNA-correlated
OS: Overall survival
NTP: Nearest template prediction
MSI: Microsatellite instability
MSS: Microsatellite stability
EBV: Epstein–Barr virus
CIMP: CpG island methylation phenotype
CIN: Chromosomal-instable
SubMap: Subclass mapping
ICP: Immune checkpoint
CNVs: Copy number variations
TIDE: Tumor immune dysfunction and exclusion
CIC: Cancer immunity cycle
SNV: Single nucleotide variants
TGF-β: Transforming growth factor b
AUC: Area under the dose–response curve
5-FU: 5-Fluorouracil
