# Supplementary material for: Epigenetically regulated gene expression profiles decipher four molecular subtypes with prognostic and therapeutic implications in gastric cancer
Source: Clin Epigenetics. 2023 Apr 15;15:64. doi: 10.1186/s13148-023-01478-w (PMC10105476; doi:10.1186/s13148-023-01478-w)
Supplement: Supplementary file 1 — Additional file1. Figure S1: Differential expression of METcor and MIRcor genes in TCGA dataset. (A) Distribution of CpG-mRNAs and miRNA-mRNA correlation coefficients in the TCGA cohort. (B) The optimal number of clusters is 4 (k+1) determined by the Bayesian information criteria (BIC) and deviance ratio plot between 2 and 5 clusters. (C) Differential analysis of METcor and MIRcor gene expression frequencies in GC subtypes. (D) Expression frequencies of METcor_high(highly expressed), METcor_low(lowly expressed) and METcor_all genes in GC subtype. (E) Expression frequencies of MIRcor_high, MIRcor_low and MIRcor_all genes in GC subtypes. ∗P < 0.05, ∗∗P < 0.01, ∗∗∗P < 0.001, ∗∗∗∗P < 0.0001. Figure S2: The METcor and MIRcor gene expression patterns of the TCGA cohort and the GEO cohorts are consistent. (A–D) The nearest template prediction (NTP) in GEO cohorts (GSE84433, GSE84437, GSE26901, GSE62254) via 2000 subtype-specific genes was in excellent agreement with the TCGA cohort. Figure S3: Several indicators reflect the tumor immune microenvironment of GC subtypes in TCGA cohort. (A–B) The immune and stromal scores of GC subtypes. (C) The single nucleotide variants (SNV) derived neoantigens scores of GC subtypes. (D) Differential analysis of pan-fibroblast transforming growth factor b (TGF-β) response signature scores. ∗P < 0.05, ∗∗P < 0.01, ∗∗∗P < 0.001. [file 13148_2023_1478_MOESM1_ESM.docx]

**Supplementary Figures**


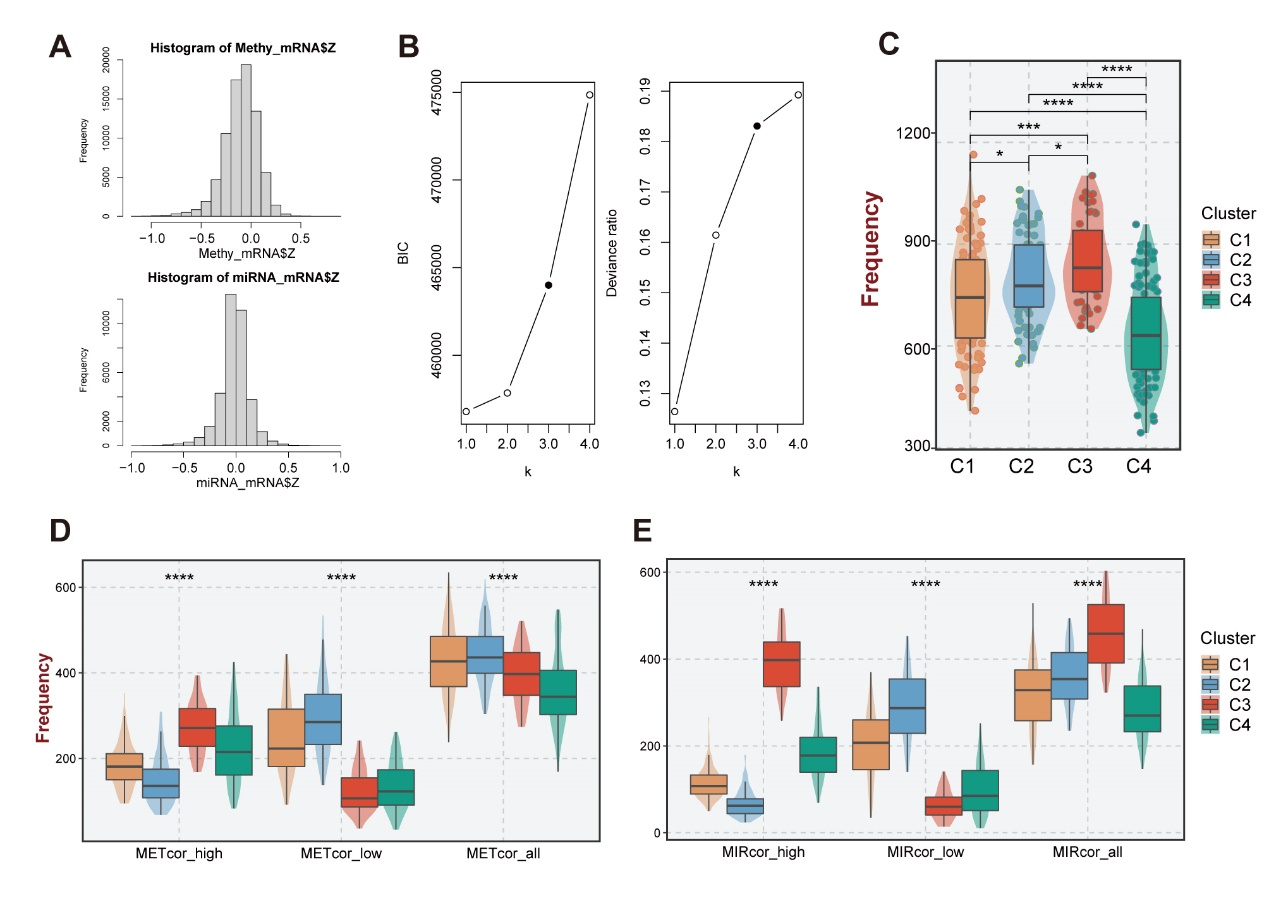


**Fig. S1** Differential expression of METcor and MIRcor genes in TCGA dataset. **(A)** Distribution of CpG-mRNAs and miRNA-mRNA correlation coefficients in the TCGA cohort. **(B)** The optimal number of clusters is 4 (k+1) determined by the Bayesian information criteria (BIC) and deviance ratio plot between 2 and 5 clusters. **(C)** Differential analysis of METcor and MIRcor gene expression frequencies in GC subtypes. **(D)** Expression frequencies of METcor_high, METcor_low and METcor_all genes in GC subtype. **(E)** Expression frequencies of MIRcor_high, MIRcor_low and MIRcor_all genes in GC subtypes. ∗*P* < 0.05, ∗∗*P* < 0.01, ∗∗∗*P* < 0.001, ∗∗∗∗*P* < 0.0001.


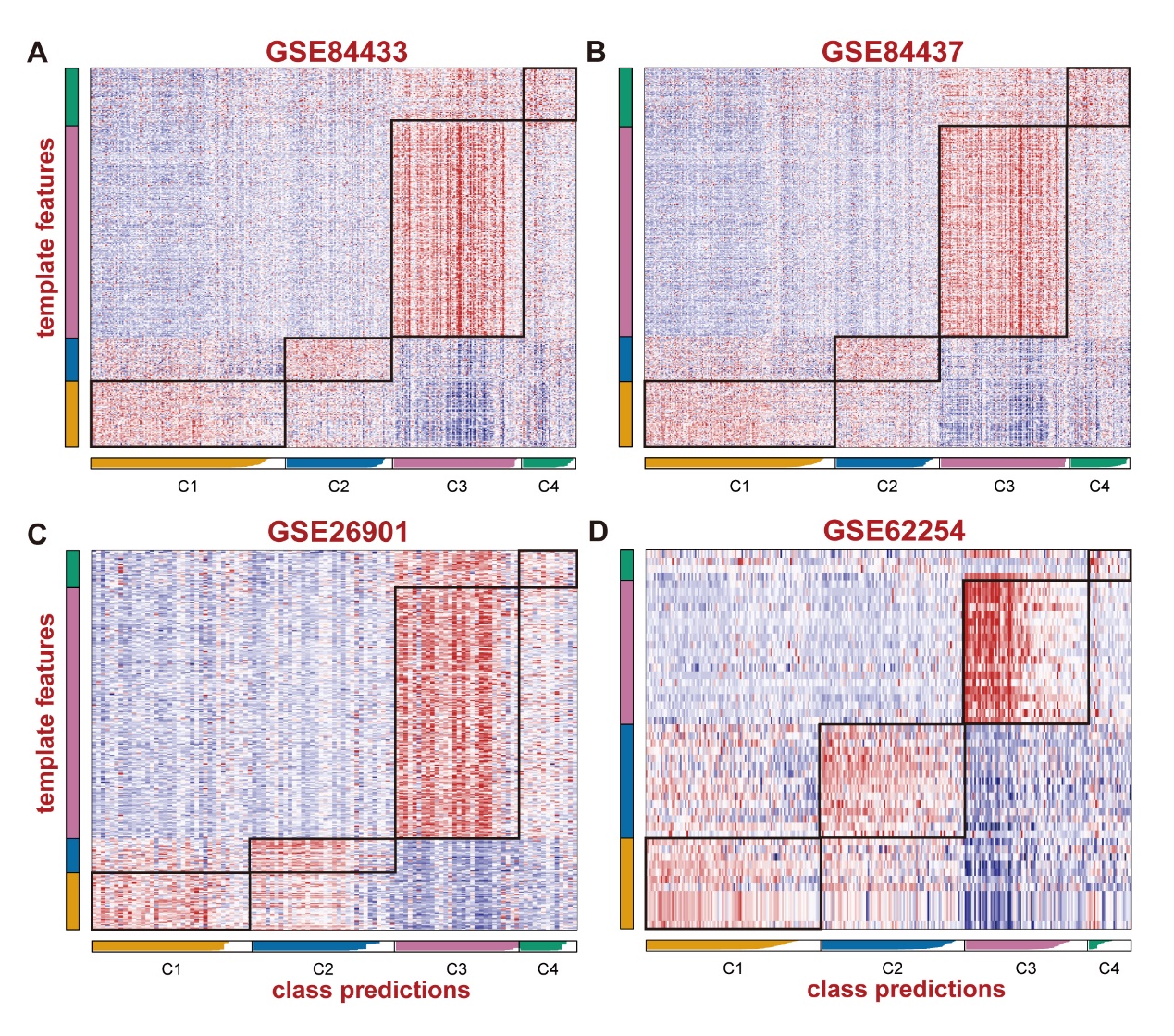


**Fig. S2** The METcor and MIRcor gene expression patterns of the TCGA cohort and the GEO cohorts are consistent. **(A-D)** The nearest template prediction (NTP) in GEO cohorts (GSE84433, GSE84437, GSE26901, GSE62254) via 2000 subtype-specific genes were in excellent agreement with the TCGA cohort.


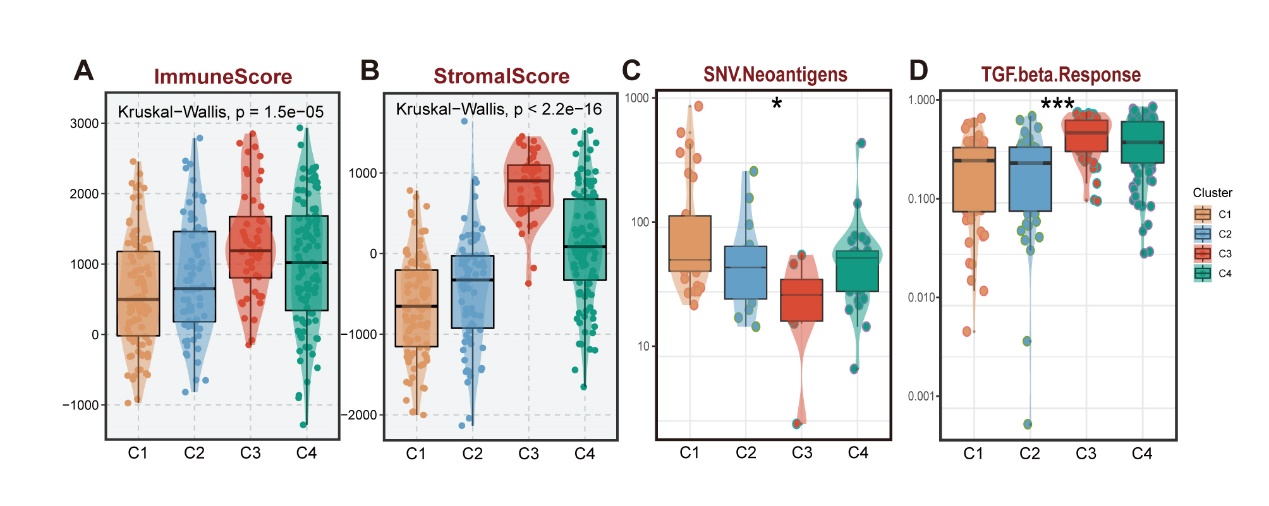


**Fig. S3** Several indicators reflect the tumor immune microenvironment of GC subtypes in TCGA cohort. **(A-B)** The immune and stromal scores of GC subtypes. **(C)** the single nucleotide variants (SNV) derived neoantigens scores of GC subtypes. **(D)** Differential analysis of pan-fibroblast transforming growth factor b (TGF-β) response signature scores. ∗*P* < 0.05, ∗∗*P* < 0.01, ∗∗∗*P* < 0.001.
